# Hairy suckers: the surface microstructure and its possible functional significance in the *Octopus vulgaris* sucker

**DOI:** 10.3762/bjnano.5.66

**Published:** 2014-05-02

**Authors:** Francesca Tramacere, Esther Appel, Barbara Mazzolai, Stanislav N Gorb

**Affiliations:** 1 Center for Micro-BioRobotics, Istituto Italiano di Tecnologia, viale Rinaldo Piaggio 34, Pontedera 56025, Italy; 2 Functional Morphology and Biomechanics, Zoological Institute, Kiel University, Am Botanischen Garten 1–9, Kiel 24098, Germany

**Keywords:** adhesion, attachment, Mollusca, octopus sucker, underwater sealing

## Abstract

Octopus suckers are able to attach to any smooth surface and many rough surfaces. Here, we have discovered that the sucker surface, which has been hypothesised to be responsible for sealing the orifice during adhesion, is not smooth as previously assumed, but is completely covered by a dense network of hair-like micro-outgrowths. This finding is particularly important because it provides another demonstration of the role of hair-structures in a sealing mechanism in water, similar to that previously described for clingfish and abalones. Moreover, the discovered hairs may provide an additional adhesive mechanism that works in concert with suction. The discovered surface structures might be potentially interesting for biomimetics of novel technical suction cups with improved adhesion capabilities on non-smooth surfaces.

## Introduction

**Figure 1:**
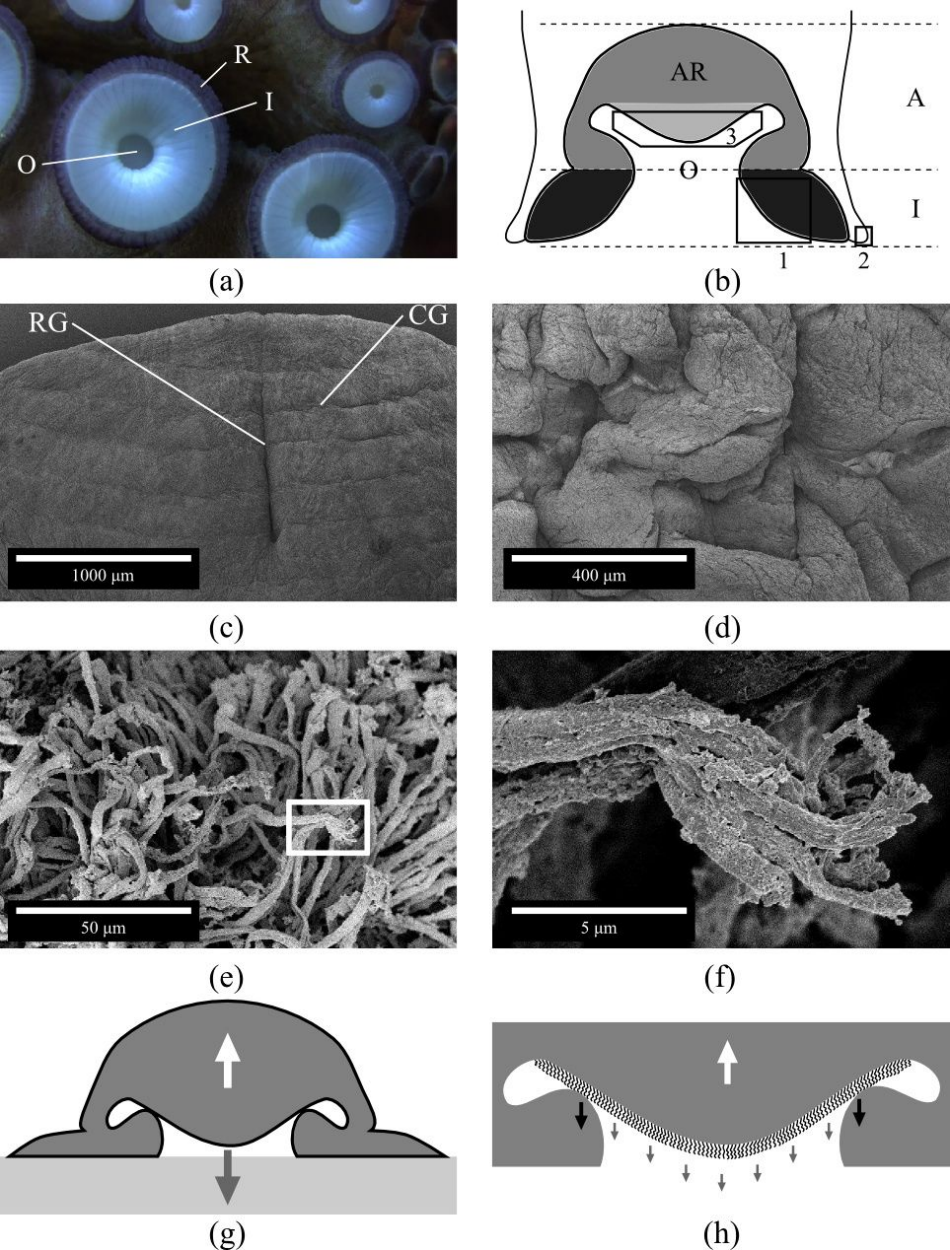
(a) Photograph of the frontal view of an octopus sucker. Infundibulum (I); orifice (O); and rim (R). (b) Schematic structure (transversal section) of an octopus sucker. The upper part in grey (light and dark grey) is the acetabulum (A). The lower disk-like black-coloured portion is the infundibulum (I). The light grey-coloured portion is the protuberance of the acetabular roof (AR); and O is the orifice. (c–f) Scanning electron microscopy images of an octopus sucker. (c) Infundibulum (please, refer to black box 1 in (b)). Radial (RG) and circumferential (CG) grooves. (d) Rim (please, refer to black box 2 in (b) and label R in (a)). (e) Surface of acetabular protuberance (please, refer to black box 3 in (b)). (f) Enlargement of white box in (e). (g) Sucker configuration during adhesion process [5]. The restoring elastic force (white arrow) is balanced by the cohesive forces of the water (grey arrow). (h) Enlargement of (g) considering the contribution of hairs. The restoring elastic force (white arrow) is counterbalanced by the cohesive forces of the water (grey arrows) and the adhesion force exerted by hairs (black arrows).

An octopus sucker consists of two portions: an upper hollow cup, the acetabulum; and a lower disk-like portion, the infundibulum, located at the attachment face of the sucker (Figure 1a,b). It is known that octopus suckers adhere not only to perfectly smooth surfaces but also to surfaces with a certain roughness [1], where technical suction cups usually fail [2–3]. This ability can be explained by the exceptional materials properties, i.e., softness, of the infundibulum [4]. However, it remains unclear how the octopus is able to remain attached to a wall or object for a long period of time [1]. Recently, it has been shown that in *Octopus vulgaris*, the acetabulum does not have a spherical shape as was previously described in the literature [1]; rather, there is a well-developed protuberance on its roof sticking out towards the orifice (Figure 1b) [5].

Additionally, it has been hypothesised that this acetabular protuberance plays a crucial role in increasing the performance of the adhesive system [5]. The pressing of the acetabular protuberance against the orifice was suggested to close the orifice when the suction is active [5]. Thus, if the suction muscles stop contracting, the maintenance of the low pressure at the interface is guaranteed by the closure of the orifice [5]. This mechanism would allow the octopus to perform an efficient attachment for a long period of time with minimal energy consumption. A specialised microstructure, as recently shown for clingfish [3] and abalone molluscs [6], might play an important watertight role in this minimal energy consumption hypothesis [5].

For studying this, we used scanning electron microscopy (SEM) at high resolution to explore the microstructure of different octopus sucker surfaces (acetabular protuberance and infundibulum). Considering the substantial interest of engineers in this biological system as a source of inspiration [7–10] for biomimetics of novel technical suction cups, our findings may provide an interesting idea for improving the adhesion capability of artificial devices on non-smooth surfaces.

## Experimental

The surfaces of the acetabular protuberance and infundibulum of *Octopus vulgaris* suckers were observed by using a Hitachi S-4800 (Hitachi High-Technologies Corp., Tokyo, Japan) scanning electron microscope. Both sexes with a body mass range from 400 to 850 g were analysed. Animals were obtained from licensed fishermen, who captured these animals from the bay of Livorno (Italy) in October 2012 for human consumption. The suckers were taken from freshly killed animals. All facilities and procedures complied with European law (Directive 2010/63/EU).

For SEM, the freshly explanted suckers were fixed in 70% ethanol. Small portions of the acetabulum and infundibulum were dehydrated and then, critical point dried by using a critical point drying apparatus (E3000 Series, Quorum Technologies, UK). Dried samples were mounted on aluminium stubs and sputter coated with a 10 nm thick layer of gold–palladium (SCD 500 sputter coater equipped with QSG 100 quartz film thickness monitor, BAL-TEC, Liechtenstein).

## Results

The arms of the octopus species examined (*Octopus vulgaris*) are characterised by two rows of suckers with diameters that range from a few centimetres (in the proximal region) to a few millimetres (in the distal region). All the suckers investigated presented the same gross/general morphology. The infundibulum (please refer to I in Figure 1a,b), which is the externally visible portion of the sucker, is entirely encircled by an epithelial rim (please refer to R in Figure 1a) and communicates with the internal portion of the sucker (please refer to A in Figure 1b) through an orifice (please refer to O in Figure 1(a,b). On the acetabular roof (please refer to AR in Figure 1b), there is a protuberance that sticks out toward the orifice (Figure 1b).

The infundibulum, which is the portion that comes in contact with the substrate during attachment, exhibits a dense network of radial and circumferential grooves on its surface (please refer to RG and CG in Figure 1c). The radial grooves appear deeper than the circumferential ones. In addition, the entire surface of the infundibulum is covered by randomly distributed, interconnected ridges. The rim that encircles the infundibulum appears like a deeply folded, loose epithelium, characterised by numerous grooves and ridges (Figure 1d).

The surface of the acetabular protuberance is completely covered with a dense network of brush-like hairs, which are approximately 50 µm (±18 µm, *n* = 25) long and have a diameter of 2 µm (±0.9 µm, *n* = 25) (Figure 1e,f). Each hair apically branches into very small filaments, which are approximately 5 µm (±2.8 µm, *n* = 18) long and have a diameter of 0.3 µm (±0.2 µm, *n* = 25) (Figure 1f). These hairy structures are only localised on the acetabular protuberance and are completely absent in the infundibular portion.

## Discussion

The surface of the infundibulum allows strong adaptation to any type of non-smooth substrate. It was observed that an octopus is able to attach to very rough surfaces generating a pressure difference of up to 0.268 MPa [11]. To obtain such an adhesion force, a perfect seal at the interface between the sucker and the substrate is crucial. Similar to abalone, in which the external surface of its side foot is characterised by many crests and grooves, in octopus suckers the seal is guaranteed by the presence of ridges and grooves on the rim encircling the infundibulum. These structures allow the octopus suckers, as well as the abalone feet, to perfectly adapt to the contour of the objects with which they come in contact.

The most important discovery of this work is the presence of hairs on the acetabular protuberance. This is of particular interest because to the best of our knowledge, these microstructures are unknown in the literature. Moreover, the presence of such hierarchical hairs on the entire surface of the acetabular protuberance supports the adhesion mechanism recently suggested for *O. vulgaris* [5]. To obtain an efficient attachment mechanism for a long period of time, the adhesion configuration shown in Figure 1g should be maintained without any muscular effort. In detail, the restoring elastic force (please refer to white arrow in Figure 1g), responsible for the detachment of the acetabular protuberance from the upper surface of the side wall of the orifice, needs to be balanced. In [5], such a force is balanced by the cohesive forces of the water in the infundibular portion (the water under tension behaves like a solid) (please refer to grey arrow in Figure 1g). Based on our recent results, an adhesion force (please refer to black arrows in Figure 1f), exerted by the dense network of hairs, is present on the surface of the acetabular protuberance. This force might work in addition to the cohesive forces of water, assisting in keeping the orifice closed for extended periods of time and significantly increasing the resistance to the restoring force. In this new scenario, the restoring elastic force is balanced by the cohesive forces of the water in the infundibular compartment and the adhesion force exerted by the hairs. In this way, the forces are in equilibrium and the sucker is able to maintain the adhesion configuration without any further energy consumption (muscular involvement). Moreover, the acetabular protuberance could work as a valve that enables and disables the adhesion process [5], and the hairs could ensure the watertight closure of the valve.

A similar hair structure was also found in the northern clingfish, *Gobiesox maeandricus* [3]. On its ventral side, this fish bears an adhesive disc that allows the animal to attach on smooth surfaces and on very rough surfaces and to resist strong water currents. The hairs structures observed in the fish are quite similar in size and aspect ratio to the hairs described here in the octopus sucker. In addition, the sole foot epithelium of abalone *Haliotis tuberculata* is characterised by a dense field of long hairs, here called cilia, that measure 0.2–0.3 µm in diameter [6]. In this case, small ciliary tufts occur at a very low density in the side foot; whereas, they become significantly more dense in the sole foot. Similar to octopus suckers, the cilia are covered by a layer of mucus. However, in all the three cases (octopus, abalone, and clingfish), the hairs lack the spatulate termini that are well known in the attachment pads of terrestrial animals [12–13]. In the case of the clingfish, it was hypothesised that the amazing tenacity observed for this fish could be related to the hierarchical structure of the hairs (“microvilli”) [3]. Moreover, the absence of spatulate termini contact elements as well as the presence of water and mucus between hairs and respective substrates suggest that biological structures operating underwater cannot exploit filament-like structures to generate van der Waals forces [3]. We completely agree with this idea and think that under wet adhesion conditions, a system consisting of hairs, mucus, and water (just like octopus suckers) could improve attachment due to following mechanisms: (i) exploiting the presence of mucus and filaments to increase the viscosity coefficient at the interface and to resist to the shear forces; and (ii) exploiting the high bulk modulus of water between the sucker and substrate that resists tensile stress (detachment force). Moreover, some studies on artificial materials have demonstrated that fibrillar microstructures are preferred to flat surfaces in applications in which a total attachment force must be generated in a binary on/off state [14]. These studies also reveal that structured surfaces show a 25% increase in pull-off force when immersed in water, and their underwater attachment is 20 times more effective than that of flat surfaces [15].

The grooves found in the infundibulum area generating a dense network of interconnected channels are instead fundamental for increasing the adhesion area subjected to the suction. Due to the presence of a particular surface microstructure, an octopus can maintain attachment for long periods of time without muscular effort and thus without energy expenditure. This functional mechanism represents a very interesting source of inspiration for engineers and robotics specialists in the development of novel biomimetic adhesion devices.

## References

[1] Kier W M, Smith A M (2002). Integr Comp Biol.

[2] Berengueres J, Tadakuma K, Kamoi T, Kratz R (2007). Compliant Distributed Magnetic Adhesion Device for Wall Climbing. Proceedings of the International Conference on Robotics and Automation.

[3] Wainwright D K, Kleinteich T, Kleinteich A, Gorb S N, Summers A P (2013). Biol Lett.

[4] Tramacere F, Kovalev A, Kleinteich T, Gorb S N, Mazzolai B (2014). J R Soc Interface.

[5] Tramacere F, Beccai L, Kuba M, Gozzi A, Bifone A, Mazzolai B (2013). PLoS One.

[6] Bravo Portela I, Martinez-Zorzano V S, Molist-Perez I, Molist García P (2012). Sci World J.

[7] Grasso F W, Setlur P (2007). Bioinspiration Biomimetics.

[8] Hu B-s, Wang L-w, Fu Z, Zhao Y-z (2009). Int J Adv Rob Syst.

[9] Tramacere F, Beccai L, Sinibaldi E, Laschi C, Mazzolai B (2011). Procedia Comput Sci.

[10] Tramacere F, Beccai L, Mattioli F, Sinibaldi E, Mazzolai B (2012). Artificial adhesion mechanisms inspired by octopus suckers. Proceedings of the International Conference on Robotics and Automation (ICRA).

[11] Smith A M (1991). J Exp Biol.

[12] Persson B N J, Gorb S (2003). J Chem Phys.

[13] Varenberg M, Pugno N M, Gorb S N (2010). Soft Matter.

[14] Varenberg M, Gorb S (2007). J R Soc Interface.

[15] Varenberg M, Gorb S (2008). J R Soc Interface.

